# 

**Published:** 1896-01

**Authors:** 


### TABLE OF CONTENTS.

ORIGINAL COMMUNICATIONS. PAGE

Electric Heat in Dental Practice. By Levitt E. Custer, B.S., D.D.S., Dayton, Ohio . . . . . . . . . . . . . . . . . . . . . . . . . . . 1

The Profession and the Man. By Rev. Edwin H. Hughes, Newton Centre, Mass. . . . . . . . . . . . . . . . . . . . . . . . . . . 9

Progressive Dentistry. By J. C. Townsend, D.D.S., Colorado Springs, Col. 17

REPORTS OF SOCIETY MEETINGS.

American Dental Association . . . . . . . . . . . . . . . . . . . . . . 23

Harvard Odontological Society . . . . . . . . . . . . . . . . . . . . . 41

Odontological Society of Pennsylvania . . . . . . . . . . . . . . . . . . 52

EDITORIAL.

Dr. G. V. Black's Investigations of the Human Teeth . . . . . . . . . . 57

BIBLIOGRAPHY . . . . . . . . . . . . . . . . . . . . . . . . . . 61

OBITUARY.

John De Haven White, M.D., D.D.S. . . . . . . . . . . . . . . . . . . 63

Resolutions of Respect to the Memory of Dr. Thomas H. Chandler . . . . 63

CURRENT NEWS.

Meeting in Western Pennsylvania . . . . . . . . . . . . . . . . . . . 64

